# Effect of a novel dwarfing mutant site on chromosome 4B on agronomic traits in common wheat

**DOI:** 10.3389/fpls.2024.1338425

**Published:** 2024-03-20

**Authors:** Jiamin Hao, Zhangchen Zhao, Xiaoge Fu, Yujiao Zhao, Muhammad Ateeq, Liming Mou, Yong Han, Yangbin Liu, Yongan Yin, Lyudmila Zotova, Dauren Serikbay, Chunyan Fan, Yin-Gang Hu, Liang Chen

**Affiliations:** ^1^ State Key Laboratory of Crop Stress Resistance and High-Efficiency Production and College of Agronomy, Northwest A&F University, Yangling, Shaanxi, China; ^2^ Dingxi Academy of Agricultural Sciences, Dingxi, Gansu, China; ^3^ Jiushenghe Seed Industry Co., Ltd, Changji, Xinjiang, China; ^4^ Yangling Digital Agricultural Technology Co., Ltd, Yangling, Shaanxi, China; ^5^ Shaanxi Grain and Agriculture Group Co., Ltd., Xi’an, Shaanxi, China; ^6^ Faculty of Agronomy, S. Seifullin Kazakh Agro Technical Research University, Astana, Kazakhstan; ^7^ Baoji Academy of Agricultural Sciences, Baoji, China; ^8^ Institute of Water Saving Agriculture in Arid Regions of China, Northwest A&F University, Yangling, Shaanxi, China

**Keywords:** dwarf gene, plant height, lodging resistance, wheat, agronomic traits

## Abstract

The introduction of dwarfing genes triggered a wave of “green revolution”. A number of wheats dwarfing genes have been reported in previous studies, and only a small fraction of these have been applied to production practices. Therefore, the development of novel dwarfing genes for wheat is of great value. In this study, a novel dwarfing site, *Rht-yz*, identified in the Yanzhan mutation, is located on chromosome 4B (30-33MB) and its mechanism of action is different from that of *Rht-B1b* (C-T mutation), but whether it affects the *Rht-B1a* (*TraesCS4B02G043100*) or other genes is unclear. Exogenously applied GA_3_ experiments showed that *Rht-yz* is one of the gibberellin-insensitive dwarf genes. The effects of the dwarf gene *Rht-yz* on agronomic traits in wheat were evaluated in the field using Yanzhan, Yanzhan mutations, F_2:3_ and F_3:4_ lines. The results showed that *Rht-yz* improved lodging resistance by reducing plant height, increasing diameter, wall thickness and mechanical strength of the basal stem. In terms of yield traits, *Rht-yz* had negative effects on tiller number plant^-1^, biomass plant^-1^ and yield plant^-1^, but had no significant effect on harvest index, 1000-kernel weight and spike traits. In addition, *Rht-yz* significantly increased crude protein, wet gluten and starch content. Therefore, the rational use of the new dwarfing site *Rht-yz* has potential and value in dwarf wheat breeding.

## Introduction

Wheat (*Triticum aestivum L.*) is an important cereal crop playing outstanding role in feeding the hunger world and global food security. Plant height is one of the main agronomic traits for morphogenesis affecting wheat yield. In the “Green Revolution” of late 20th century, *Rht-B1b* and *Rht-D1b* significantly reduced plant height in wheat and increased wheat production (Hedden, 2003). Wheat varieties carrying dwarf genes *Rht-B1b* and *Rht-D1b* (Chihoumu and Dharma) rapidly expanded the planting area, and the wheat dwarf gene was gradually used in wheat breeding. To date, more than 25 dwarf genes have been discovered. The mainly used wheat dwarf genes were *Rht-B1b*, *Rht-D1b* and *Rht8*, which limited the improvement in wheat yield potential. *Rht-B1b*/*Rht-D1b* reduced plant height by 23%, and *Rht-B1b*+*Rht-D1b* reduced plant height by 47% (Richards, 1992), but *Rht-B1b* and *Rht-D1b* had certain adverse effects on seedling traits. The dwarf gene *Rht-B1b* and *Rht8* also had a significant effect on the tillers number plant^-1^ in wheat. *Rht-B1b* reduced 1000-kernel weight but increased yield to eliminate the negative effect of small biomass (Hayat et al., 2019). Meanwhile, *Rht-B1b* and *Rht-D1b* associated with reduction in seed size and protein content (Casebow et al., 2016).

Furthermore, the dwarf gene *Rht3* has a three times greater effect on plant height than *Rht-B1b*, but plants that are too short are not conducive to yield formation (Gale et al., 1985; Flintham et al., 2000; Derkx et al., 2017). *Rht5* reduced plant height by 25.84%, but had a negative effect on spike development (Daoura et al., 2014). *Rht13* reduces plant height by 21.5% but has a negative effect on internode diameter (Wang et al., 2014). Therefore, many dwarf genes are difficult to be applied in wheat breeding.

Statistics show that about 70% of the wheat varieties released worldwide contain *Rht-B1b* or/and *Rht-D1b* (Evans, 2008). During 2012-2018, *Rht-D1b* was present at a frequency of more than 80% in Uniform Southern Soft Red Winter Wheat Nursery (Hayat et al., 2019). In addition, more than half of breeding lines in SRWW in the southern United States carry *Rht-D1b*, and approximately 31% of breeding lines carry *Rht-B1b* (Guedira et al., 2010). *Rht-B1b* and *Rht-D1b* were also widely distributed in Chinese wheat, the genetic frequencies of *Rht-D1b*, *Rht8* and *Rht-B1b* were 45.5%, 46.8% and 24.5%, respectively (Zhang et al., 2006).

However, the widespread distribution of single dwarf source is not beneficial to the sustainable development of wheat breeding, which reduces the diversity of dwarf sources and the safety of wheat production. Wheat dwarf breeding requires diversified dwarf genes. Therefore, it is important to identify new wheat dwarf genes and determine their application value for wheat dwarf breeding. The aim of this study was to identify the novel wheat dwarfing site *Rht-yz*, and to clarify its effects on wheat agronomic traits by measuring plant height, internode traits, lodging resistance traits, yield and grain traits.

## Materials and methods

### Materials and field experiment

The Yanzhan 4110 (followed as Yanzhan) and natural dwarf mutants of Yanzhan 4110 (followed as Yanzhan mutation) provided by Dong Puhui’s research group at Henan University of Science and Technology (HAUST), were crossed to produce F_2:3_ and F_3:4_ populations, including 15 dwarf lines and 15 tall lines. Field trials were conducted in 2019-2020 and 2020-2021 at the experimental farm of the Institute of Water-Saving Agriculture for Arid Areas of China, Northwest A&F University, Yangling, Shaanxi, China. The experimental design was randomized complete block design (RCBD), and each line was planted in 2.5m^2^ sized plots (five rows of 2 m long, 25 cm between two rows, and 6.7 cm between two plants). Each plot was repeated three times.

The GA_3_ treatment was applied separately on both Yanzhan and Yanzhan mutation, and repeated three times. When wheat plants reached at stem elongation (Z31), early heading (Z51), early flowering (Z61), and grain filling (Z71) stages, GA_3_ solution (100 μM) was sprayed on the surfaces of leaves, culms and young panicles (Zadoks et al., 1974), and the control was applied with distilled water of the same volume. GA_3_ treatments in field were performed referred to Duan et al. 2020a.

### Genomic sequence clone

Genomic DNA was extracted by CTAB method (Clark, 1997). The genomic sequence of *Rht-B1b* was cloned with primers in **Supplementary Table 1**, using PCR conditions described. PCR was performed in a Peltier Thermal cycler with a 20μl volume used ApexHF HS DNA Polymerase FL (Accurate Biology, China), and the PCR program was set according to the instructions (temperature was 60°C, extension time was 3min). PCR products were distinguished by 2% agarose gel electrophoresis.

The PCR target band was purified using Universal DNA Purification Kit (Tiangen, China). After adding ‘A’ base at the end, the recovered product was cloned into the T vector (TAKARA pMD18-T Vector Cloning Kit). The vector was transferred into E. coli DH5α competent cells by the 42°C heat shock method, and 950μl of SOC liquid medium was added to the 37°C shaking culture for 45 minutes. The product was plated in AMP-containing LB medium. After overnight incubation at 37°C, individual colonies were detected and sequenced. Primer synthesis and clone sequencing were performed at Tsingke Biology Technique (Beijing, China).

### DNA extraction based on bulked segregation analysis

Yanzhan and Yanzhan mutation F_2_ population used to create tall and dwarf mixed pools. Genomic DNA was extracted from the second leaf below the spike of 20 tall and 20 dwarf plants at the filling stage. Equal amounts of tall plant genomic DNA were mixed into tall mixed pools, and equal amounts of dwarf plants genomic DNA were mixed into dwarf mixed pools. Combined with 660K chip (Affymetrix), SNP typing was detected (CapitalBio Technology, Beijing, China).

### Plant height and internode length traits

Plant height and the internode length were investigated at the mature stage. Internode diameter and wall thickness were measured with Vernier scale during the grain filling stage (Du et al., 2018), with five replicates.

With reference to the method of Zhao et al. (2021), the internodes strength tester was used to measure the mechanical strength of the 4th and 5th basal internodes. The center of gravity height and fresh weight of the main stem were also measured. The formula for calculating the lodging resistance index is as follows:


LRI=MS÷(CGH ×FW)

LRI: Lodging Resistance Index; MS: Mechanical Strength (N); CGH: Center of Gravity Height (m); FW: Fresh Weight(g).

### Yield traits

The standard and method for yield characters were referred to “Physiological Breeding II: A field Guide to Wheat Phenotyping” (Reynolds, 2012). At maturity, tillers plant^-1^, spike length, spikelet number spike^-1^, grains number spike^-1^, 1000-kernel weight, biomass plant^-1^, yield plant^-1^ and harvest index were measured using a random sample of 20 individual plants.

### Grain traits

After threshing and drying, grain length, width and diameter were measured using the SC-G automatic seed analyzer (Wanshen, China). Grain quality traits, including water content, crude protein, wet gluten, water absorption, starch, stable time and formation time were measured using a Diode Array 7250 near-infrared reflectance (NIR) spectrometer (Perten Instrument AB, Sweden).

### Data analysis

The formula for the difference between dwarf and tall plants as follow:


$$\text{Difference }\left(\%\right)\;=\;\left({\text{Value}}_{\text{Dwarf}}-{\text{Value}}_{\text{Tall}}\right)÷{\text{ Value}}_{\text{Tall}}\times \;100\%$$

Data was analyzed using analysis of variance (ANOVA) for a t-test of the difference between the dwarf and tall lines using IBM SPSS Statistics 23.0. * means significant at p< 0.05. ** means significant at p< 0.01, none means not significant at p > 0.05. Oligo 7 was used for primer design. Origin 2018 and Microsoft Office Excel 2016 were used to complete the figures and tables.

## Results

### Discovery of a novel dwarfing site

#### Exogenous GA_3_ treatment of Yanzhan and Yanzhan mutation

Compared to Yanzhan, the height of the mutant was reduced by 22.5 cm (29.8%). At the jointing stage, Yanzhan and Yanzhan mutants were treated with exogenous GA_3_. The results showed that exogenous GA_3_ had no significant effect on plant height, length, diameter and wall thickness of the fourth and fifth stems of Yanzhan and Yanzhan mutants (**Table 1**). This indicated that the type of Yanzhan mutation was gibberellin insensitive and the mutation was not responsive to exogenous gibberellin.

**Table 1:** Effect of exogenous GA_3_ treatment on plant height and internodes traits of YZ and M.

Phenotypes		YZ	YZ-GA_3_	Difference	M	M-GA_3_	Difference
**Plant height (cm)**	Plant height Range (95%)	75.4±0.49 74.3~76.6	74.6±0.43 73.6~75.6	-0.8 (-1.1%)	52.9±0.45 51.9~53.9	54.7±0.45 53.7~55.8	1.8 (3.4%)
**Internode length (cm)**	I4 Range (95%)	7.5±0.12 7.2~7.8	7.5±0.13 7.0~7.9	0.0	4.8±0.26 4.1~5.4	5.4±0.27 4.6~6.3	0.6 (12.5%)
	I5 Range (95%)	7.1±0.31 6.3~7.8	8.2±0.06 7.9~8.4	1.1 (15.5%)	3.1±0.14 2.7~3.5	3.5±0.25 3.2~4.7	0.4 (12.9%)
**Internode diameter (mm)**	I4 Range (95%)	4.3±0.03 4.3~4.4	4.3±0.04 4.2~4.4	0.0	4.6±0.04 4.5~4.7	4.7±0.05 4.6~4.8	0.1 (2.2%)
	I5 Range (95%)	4.5±0.03 4.4~4.5	4.3±0.03 4.3~4.4	-0.2 (-4.4%)	4.5±0.09 4.1~4.4	4.4±0.09 4.3~4.6	-0.1 (-2.3%)
**Internode thickness (mm)**	I4 Range (95%)	0.8±0.03 0.7~0.8	0.7±0.02 0.7~0.8	-0.1 (-12.5%)	1.0±0.05 0.9~1.1	1.0±0.03 0.9~1.1	0.0
	I5 Range (95%)	0.8±0.03 0.7~0.9	0.8±0.02 0.7~0.8	0.0	1.1±0.05 0.9~1.1	1.1±0.04 1.2~1.3	0.0

YZ, Yanzhan; M, Yanzhan mutation. The data is mean ± SE (standard error). Different is effect of exogenous GA_3_. Difference = (Treatment – Control)/Control*100%.

#### BSA (bulked segregation analysis) of F_2_ population

**Figure 1:**
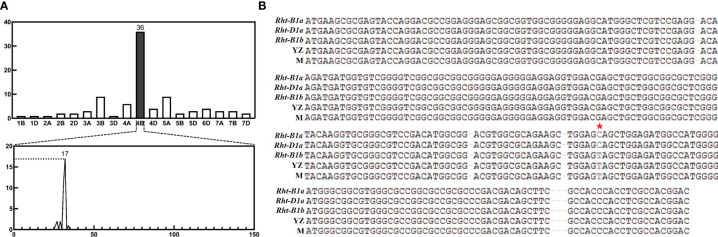
Bulked segregation analysis (BSA) results and sequencing of *Rht-yz*. **(A)** Results of BSA analysis in F_2_ population. The 4B chromosome has 36 SNPs, which concentrates on 30-33MB. **(B)** Results of *Rht-B1b* sequence. The * pointss the mutant site of *Rht-B1b*, which had no different between Yanzhan and mutation lines. YZ, Yanzhan; M, Yanzhan mutation.

The homozygous differential SNPs of dwarf and tall bulks were analyzed using BSA and 660K SNP array. The results showed that 92 high quality SNPs were detected, of which 36 (39.1%) were found on chromosome 4B, and the rest were distributed on other chromosomes. And the differential SNPs on chromosome 4B were mainly enriched on the short arm, of which 28 (77.8%) were within 30-33 Mb (**Figure 1A**). This indicated that the mutation causing dwarf plant height was likely to be located in this interval.

#### Genomic sequence clone of *Rht-B1b*

The reported dwarfing gene *Rht-B1b* was found within this interval by searching for high confidence genes in 4BS: 30-33 MB according to IWGSC RefSeq v1.1, in combination with gene annotation (**Table 2**). In order to determine the genomic sequence of *Rht-B1b*, primers were designed (**Supplementary Table 1**). The coding region sequence was cloned using PCR technology and monoclonal sequencing. The sequence alignment revealed that the mutation site of *Rht-B1b* was the same in Yanzhan and Yanzhan mutation, this indicated that the mechanism of plant height reduction of this dwarfing site may be different from that of *Rht-B1b* (C-T mutation) (**Figure 1B**).

**Table 2:** Names of dwarf genes and positional information of chromosomes.

Name	Chromosome position
*Rht1*(*RhtB1b*)	4BS
*Rht2(RhtD1b)*	4DS
*Rht3*(*RhtB1c*)	4BS
*Rht4*	2BL
*Rht5*	3BS
*Rht6*	4D
*Rht7*	2AS
*Rht8*	2DS
*Rht9*	5AL
*Rht10* (*RhtD1c*)	4DS
*Rht11* (*RhtB1e*)	4BS
*Rht12*	5AL
*Rht13*	7BS
*Rht14*	6AS
*Rht15*	6A
*Rht16*	6AS
*Rht17 (RhtB1p)*	4BS
*Rht18*	6AS
*Rht19*	–
*Rht20*	–
*Rht21*	2A
*Rht22*	7AS
*Rht23*	5DL
*Rht24*	6AL
*Rht25*	6AS

### Effect of *Rht-yz* on plant height and internodes traits

**Figure 2:**
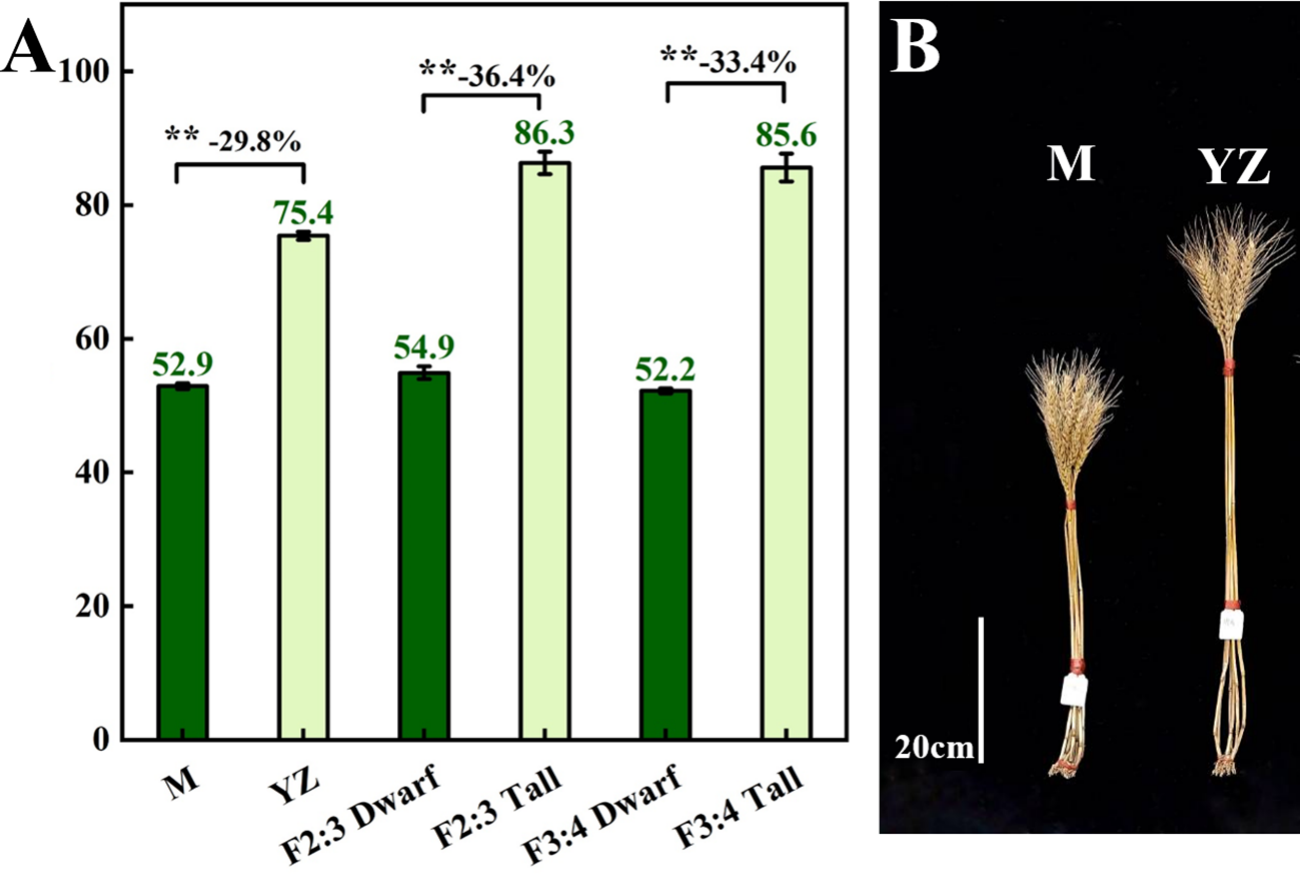
Plant height of Yanzhan mutation population. **(A)** Difference of plant height between dwarf and tall lines. **(B)** The mature plant height of Yanzhan and Yanzhan mutation. ** significant at p<0.01.

**Figure 3:**
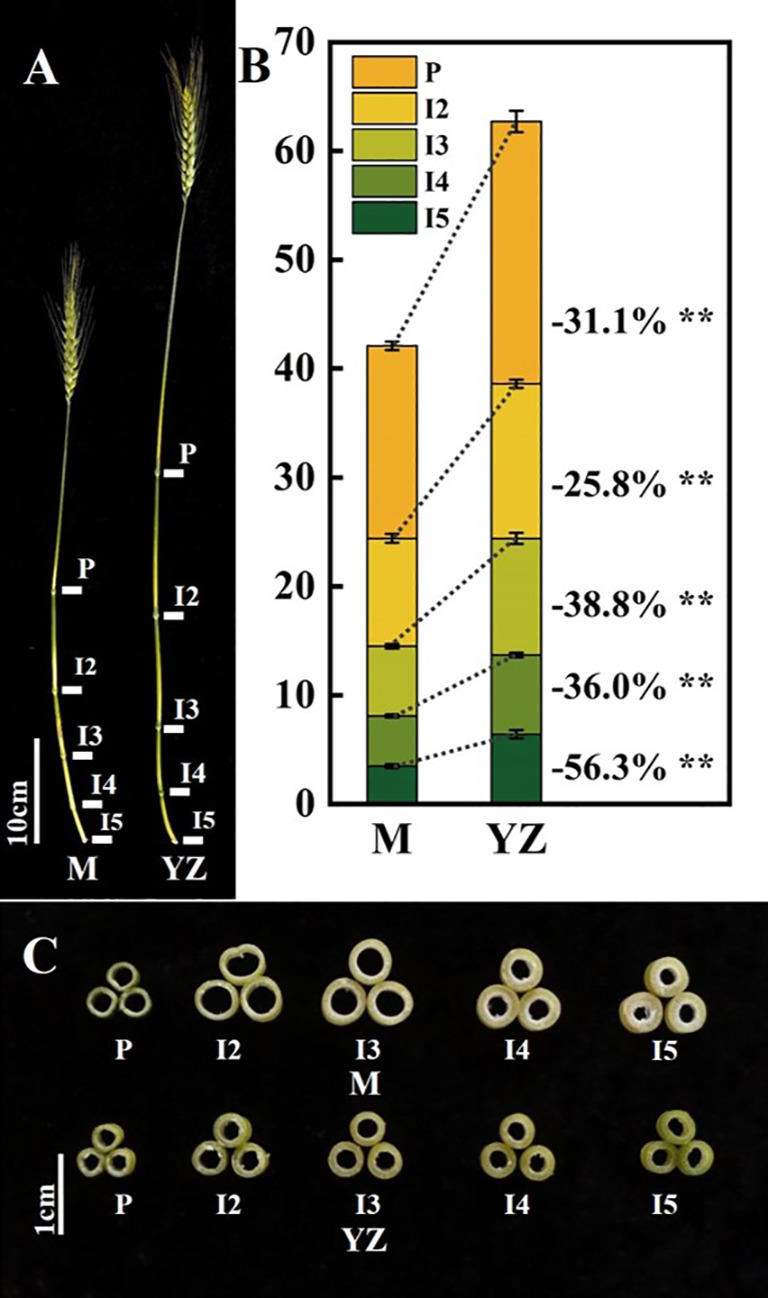
Internodes traits of Yanzhan and Yanzhan mutation at the filling stage. **(A)** The main stem. **(B)** Difference of internode length. **(C)** Cross section of the middle of each internode. ** significant at p<0.01. P, peduncle. I2, I3, I4 and I5, the second internode under spike, the third internode under spike, the fourth internode under spike, the fifth internode under spike.

*Rht-yz* significantly reduced plant height. The height of Yanzhan was approximately 75.4 cm, and the height of the Yanzhan mutant carrying *Rht-yz* was reduced by 22.5 cm (29.8%). In addition, *Rht-yz* reduced plant height by 36.4% and 33.4% in the F_2:3_ and F_3:4_ populations, respectively (**Figure 2**). *Rht-yz* also had significant effects on internode length, diameter and thickness in wheat. Both Yanzhan and Yanzhan mutations had 5 internodes, and the Yanzhan mutations had significantly shorter internode lengths compared with Yanzhan (**Figures 3A, B**), in which the peduncle was significantly shortened by 8.0 cm (31.3%), I2, I3, I4 and I5 were significantly shortened by 3.9 cm (25.8%), 4.5 cm (38.8%), 2.7 cm (36.0%) and 4.0 cm (56.3%), respectively. In addition, compared with Yanzhan the diameters of I2, I3, I4, and I5 with Yanzhan mutations were significantly increased by 0.4 mm (9.8%), 0.4 mm (9.1%), 0.3 mm (7.0%), and 0.2 mm (4.4%), respectively, the thickness of I3, I4 and I5 was significantly increased by 0.2 mm (33.3%), 0.2 mm (25.0%) and 0.3 mm (37.5%) respectively (**Figure 3C** and **Table 3**).

**Table 3:** Effect of *Rht-yz* on internode traits.

Internode traits		M	YZ	Difference	F_2:3_ dwarf	F_2:3_ tall	Difference	F_3:4_ dwarf	F_3:4_ tall	Difference
**Length (cm)**	**P**	17.7±0.58	25.7±0.42	-8.0 (-31.1%)**	16.9±0.47	27.7±0.73	-10.8 (-39.0%)**	18.0±0.69	28.1±1.03	-10.1 (-35.9%) **
	**I2**	11.2±0.28	15.1±0.44	-3.9 (-25.8%)**	8.8±0.18	17.4±0.22	-8.6 (-49.4%)**	9.7±0.33	14.3±0.35	-4.6 (-32.2%) **
	**I3**	7.1±0.10	11.6±0.11	-4.5 (-38.8%)**	6.3±0.08	13.1±0.35	-6.8 (-51.8%)**	6.4±0.26	13.9±0.51	-7.5 (-54.0%) **
	**I4**	4.8±0.26	7.5±0.12	-2.7 (-36.0%)**	4.6±0.23	8.4±0.44	-3.8 (-45.2%)**	4.5±0.37	8.1±0.22	-3.6 (-44.4%) **
	**I5**	3.1±0.14	7.1±0.31	-4.0 (-56.3%)**	3.9±0.24	6.4±0.22	-2.5 (-38.4%)**	4.0±0.19	6.5±0.44	-2.5 (-38.5%) **
**Diameter (cm)**	**P**	3.3±0.03	3.4±0.03	-0.1 (-2.9%)	2.9±0.04	2.7±0.03	0.2 (7.4%)**	2.7±0.03	2.6±0.04	0.1 (3.8%)
	**I2**	4.5±0.05	4.1±0.05	0.4 (9.8%)*	3.8±0.04	3.6±0.04	0.2 (5.6%)**	4.0±0.05	3.7±0.05	0.3 (8.1%)*
	**I3**	4.8±0.04	4.4±0.05	0.4 (9.1%)*	4.1±0.04	4.1±0.05	0.0	4.3±0.03	4.1±0.05	0.2 (4.9%)
	**I4**	4.6±0.04	4.3±0.03	0.3 (7.0%)*	4.1±0.03	4.0±0.05	0.1 (2.5%)	4.2±0.06	4.0±0.04	0.2 (5.0%)*
	**15**	4.5±0.09	4.3±0.03	0.2 (4.4%)*	4.1±0.05	3.9±0.05	0.2 (5.1%)*	4.0±0.05	3.8±0.05	0.2 (5.3%)*
**Thickness (cm)**	**P**	0.4±0.01	0.4±0.01	0.0	0.3±0.01	0.3±0.01	0.0	0.3±0.03	0.3±0.01	0.0
	**I2**	0.5±0.02	0.5±0.02	0.0	0.4±0.01	0.4±0.02	0.0	0.4±0.02	0.4±0.01	0.0
	**I3**	0.8±0.02	0.6±0.03	0.2 (33.3%)**	0.5±0.01	0.4±0.01	0.1 (25.0%) **	0.5±0.01	0.4±0.02	0.1 (25.0%)**
	**I4**	1.0±0.05	0.8±0.03	0.2 (25.0%)**	0.6±0.02	0.5±0.01	0.1 (20.0%) **	0.6±0.01	0.5±0.02	0.1 (20.0%)**
	**I5**	1.1±0.05	0.8±0.03	0.3 (37.5%)**	0.7±0.02	0.5±0.01	0.2 (40.0%) **	0.6±0.03	0.5±0.03	0.1 (20.0%)**

YZ, Yanzhan; M, Yanzhan mutation. The data is mean ± SE (Standard Error). * significant at p< 0.05; ** significant at p<0.01. P, peduncle. I2, I3, I4 and I5, the second internode under spike, the third internode under spike, the forth internode under spike, the fifth internode under spike.

Similar results were observed in the F_2:3_ and F_3:4_ populations, where *Rht-yz* significantly reduced plant height and internode length. *Rht-yz* also significantly increased the diameter of I2 and I5 and the wall thickness of I3, I4 and I5 (**Table 3**).

### Effect of *Rht-yz* on lodging resistant traits

**Figure 4:**
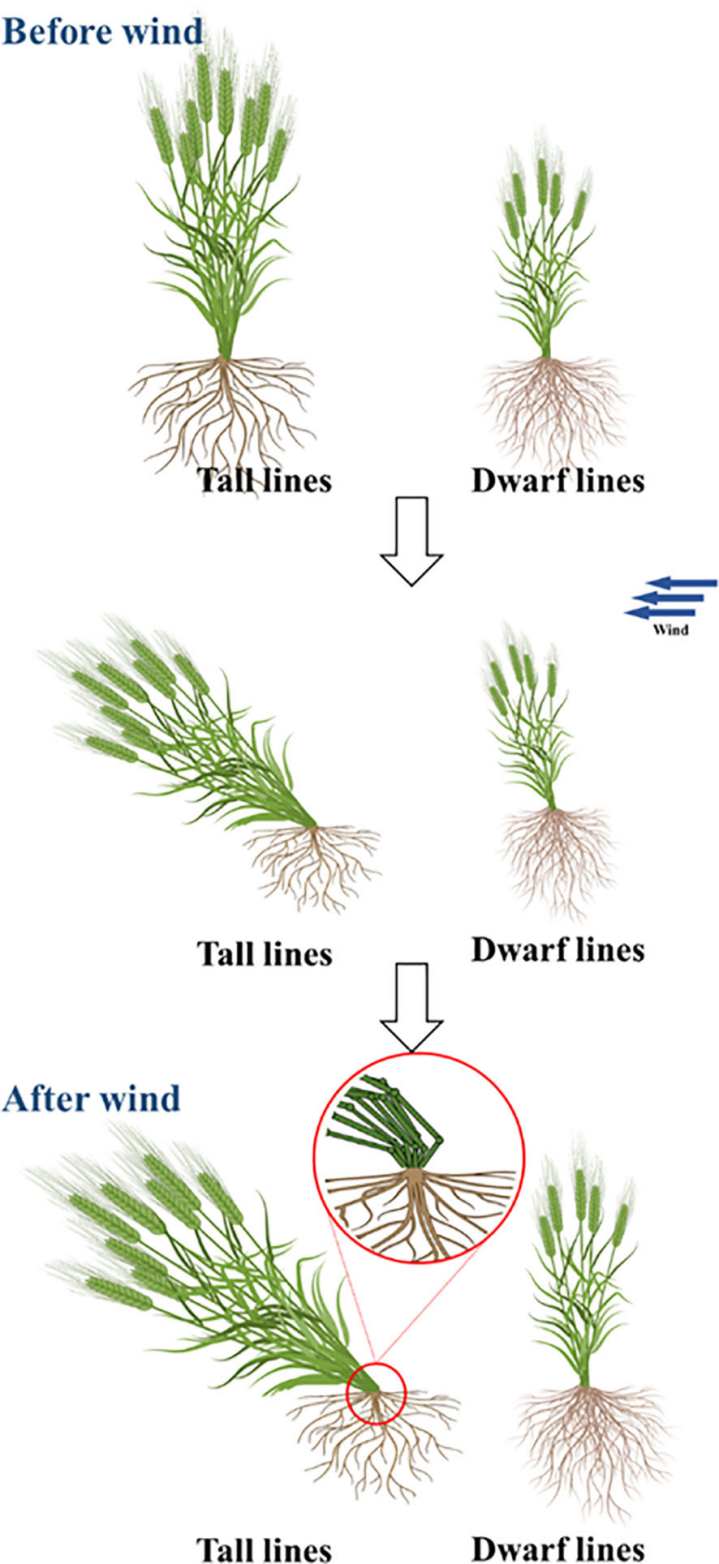
Diagram of lodging resistance of dwarf mutant.

This study focused on the lodging resistance index of the basal stem (I4 and I5), which are most relevant for lodging resistance traits (**Figure 4**). The results showed that *Rht-yz* significantly increased the mechanical strength of Yanzhan mutants I4 and I5 by 7.4% and 15.5% respectively. Meanwhile, *Rht-yz* significantly increased the lodging resistance index of I4 (72.7%) and I5 (90.6%) (**Table 4**).

**Table 4:** Effect of *Rht-yz* on internodes lodging resistance index of basal internodes.

Materials	Breaking strength (N)		Internodes lodging resistance index	
	I4	I5	I4	I5
**M**	24.8 ± 2.51	32.9 ± 2.63	7.6 ± 0.62	10.1 ± 0.83
**YZ**	23.1 ± 2.24	27.7 ± 3.14	4.4 ± 0.30	5.3 ± 0.42
**Difference**	1.7(7.4%) *	4.3(15.5%) *	3.2(72.7%) **	4.8(90.6%) **

The data is mean ± SE (Standard Error). * significant at p< 0.05; ** significant at p<0.01.

### Effect of *Rht-yz* on yield and yield component

In the parental materials, F_2:3_ and F_3:4_ populations, *Rht-yz* significantly reduced the tillers plant^-1^, biomass plant^-1^ and yield plant^-1^. In addition, 1000-kernel weight and harvest index of the Yanzhan mutant and *Rht-yz* dwarf lines were also significantly reduced in the F_2:3_ population. For spike traits, *Rht-yz* increased spike length, spikelet number spike^-1^ and grain number spike^-1^, but the difference was not significant (**Table 5**).

**Table 5:** Effect of *Rht-yz* on yield traits.

Genotype	M	YZ	Difference	F_2:3_ dwarf	F_2:3_ tall	Difference	F_3:4_ dwarf	F_3:4_ tall	Difference
**Tillers plant^-1^**	6.5±0.43	9.0±0.82	-2.5 (-27.7%) **	5.8±0.83	7.6±1.43	-1.8 (-23.6%) *	7.9±0.45	11.4±0.45	-3.5 (-30.7%) **
**Spike Length (cm)**	9.7±0.35	9.4±0.21	0.3 (3.2%)	9.9±0.17	9.7±0.20	0.2 (2.4%)	9.7±0.51	10.0±0.27	-0.3 (-3.0%)
**Spikelet number plant^-1^**	23.9±0.93	21.2 ± 1.60	2.7 (12.7%)	19.6±0.45	17.6±0.40	2.0 (11.4%)	22.9±0.18	24.6±0.34	-1.7 (-6.9%)
**Grain number plant^-1^**	38.5±0.96	34.0±1.69	4.5 (13.2%)	33.5±1.40	34.2±1.15	-0.7 (-2.2%)	37.5±1.80	38.0±1.92	-0.5 (-1.3%)
**1000-kernel weight (g)**	39.2±3.50	43.3±4.26	-4.1 (-9.5%)	32.3±1.54	40.4±3.81	-8.1 (-20.1%)	43.9±0.53	48.2±1.22	-4.3 (-8.9%)
**Biomass plant^-1^ (g)**	20.2±1.05	34.8±3.24	-14.6 (-42.0%) **	22.7±0.60	36.2±2.09	-13.5 (-37.2%) **	30.3±2.68	42.4±4.23	-12.1 (-28.5%) **
**Yield plant^-1^ (g)**	7.1±0.60	12.5±1.55	-5.4 (-43.2%) **	6.4±0.37	13.3±1.00	-6.9 (-52.0%) **	9.3±1.15	13.8±1.46	-4.5 (-32.6%) **
**Harvest index**	0.35±0.02	0.39±0.01	-0.04 (-10.25%)	0.26±0.01	0.37±0.02	-0.1 (-29.6%) **	0.31±0.03	0.33±0.02	-0.02 (-6.1%)

The data is mean ± SE (Standard Error). * significant at p< 0.05; ** significant at p<0.01.

### Effect of *Rht-yz* on grain size and quality

**Figure 5:**
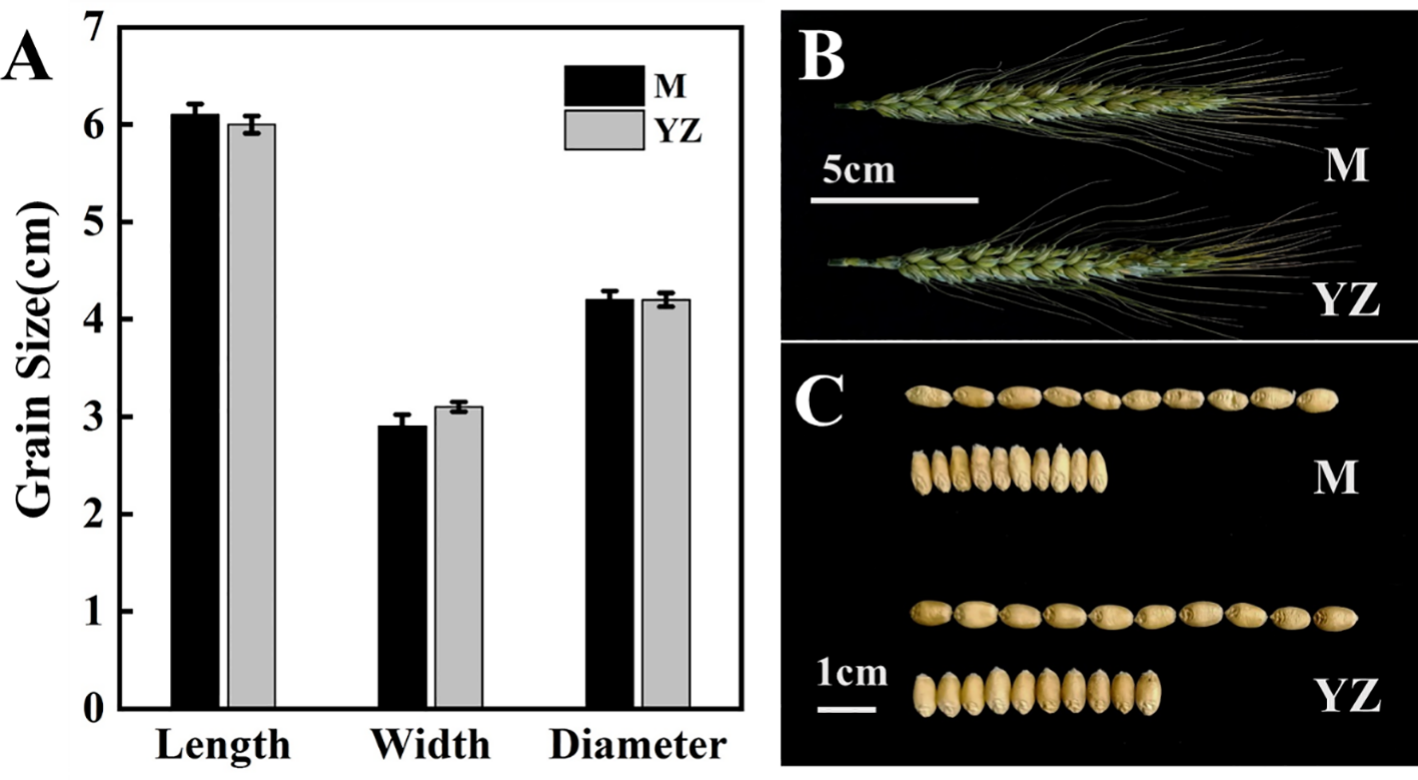
Grain traits of Yanzhan and Yanzhan mutation. **(A)** Difference of grain length, width and diameter. **(B)** Spike at the filling stage. **(C)** Grain photograph.

Grain length, width and diameter of the Yanzhan mutation were not significantly different from those of Yanzhan, but *Rht-yz* reduced grain width by 6.5% and also had a negative effect on grain plumpness (**Figure 5**).

*Rht-yz* significantly increased grain crude protein (12.8%), wet gluten (10.0%), starch (6.9%), dough stability time (70.9%) and dough developing time (7.4%), but had no significant effect on water content and water absorption (**Table 6**).

**Table 6:** Effect of *Rht-yz* on grain quality.

Traits	M	YZ	Difference
**Water content (%)**	11.4±0.01	11.4±0.01	0.0
**Crude protein (%)**	15.0±0.17	13.3±0.06	1.7(12.4%)**
**Wet gluten (%)**	31.9±0.08	29.0±0.05	3.0(10.2%)**
**Water absorption (%)**	67.4±0.06	66.3±0.03	1.1(1.7%)
**Starch (%)**	60.4±0.31	56.5±0.20	3.8(6.8%)**
**Dough stability time (min)**	13.5±0.09	7.9±0.09	5.6(71.2%)**
**Dough developing time (min)**	2.9±0.03	2.7±0.03	0.2(8.2%)**

The data is mean ± SE (Standard Error). ** significant at p<0.01.

## Discussion

The dwarfing gene solved the lodging problem in wheat breeding and greatly increased wheat yield per unit area (Flintham et al., 2000; Hedden, 2003). However, the negative effects of many dwarfing genes on yield and other traits in wheat have led to difficulties in their application. In this study, a new dwarf wheat mutant was identified and found to carry a new dwarf gene, provisionally named *Rht-yz*. To clarify the genetic effects of this gene, the plant height and internode length, lodging resistance, yield and grain traits were investigated from Yanzhan, Yanzhan mutations, F_2:3_ and F_3:4_ populations.

### Discovery of a novel dwarfing site

*Rht-B1a* is wild-type and encodes a normal DELLA protein. The *Rht-B1b* and *Rht-D1b* have single base mutations in the coding region of the DELLA protein (*Rht-B1b* has a C-T mutation and *Rht-D1b* has a G-T mutation in the coding region), which ultimately leads to inactivity of the DELLA protein and reduced plant height (Peng et al., 1999; Pearce et al., 2011). The *Rht-B1c* transcript has a 90bp insertion, while *Rht-B1d* and *Rht-B1e* are prematurely terminated by single base (AT) mutations (Pearce et al., 2011), while *Rht-B1k* has displacement mutations in the VHIID domain to produce abnormal DELLA proteins. In this study, it was found that the number of differential SNPs enriched in the chromosome 4B was higher (36) and that there were 28 differential SNPs in the 30-33 MB interval, forming a differential SNPs enrichment peak. However, the number of differential SNPs was less and the distribution was scattered on other chromosomes, which did not form an obvious differential SNPs enrichment peak. Therefore, we can only obtain one interval (4B: 30-33MB) that regulates plant height, which contains a dwarf gene, *Rht-B1b*. The mutation site of the *Rht-B1b* did not differ between the Yanzhan and Yanzhan mutations. Therefore, *Rht-yz* is a new dwarfing site that regulates plant height and its mechanism of action is different from that of *Rht-B1b* (C-T mutation), but it is not clear whether it affects the *Rht-B1a* (*TraesCS4B02G043100*) or other genes.

### *Rht-yz* improved the lodging resistance in wheat

Improving lodging resistance in wheat is one of the most important factors in increasing wheat yields. Many pre-Green Revolution wheat varieties were replaced due to lodging resistance defects. The wheat dwarf genes *Rht-B1b* and *Rht-D1b* reduced plant height by 23%, *Rht-B1b*+*Rht-D1b* reduced plant height by 50% (Butler et al., 2005). The dwarfing genes *Rht8* and *Rht12* reduced plant height by 8.0% and 40%, respectively (Tang et al., 2009; Chen et al., 2013). In this study, *Rht-yz* reduced plant height by approximately 29.8%, which was a moderate strength effect.

Studies have shown that the length of I4 and I5 is significantly correlated with lodging resistance in wheat (Kashiwagi et al., 2005), and lodging resistance is positively correlated with internode diameter, thickness and fullness (Zhao et al., 2021). Studies have shown that *Rht18* increased basal internode strength (Yang et al., 2015), while *Rht13* decreased basal internode diameter (Wang et al., 2014). In addition, the height of center of gravity is also an important factor in the lodging resistance index. In this study, *Rht-yz* mainly improved the lodging resistance by reducing the plant height and increasing the diameter, wall thickness and mechanical strength of the basal internode, which has important application value for dwarf breeding in wheat.

### *Rht-yz* had negative effect on part of the yield components

The study reported that *Rht3* increased tiller number (Zhang et al., 1995), *Rht5* reduced spikelet number and grain number spike^-1^ in two different genetic backgrounds (Daoura et al., 2014), and *Rht8* had little effect on yield traits (Rebetzke et al., 2012). In this study, *Rht-yz* had a significant negative effect on tiller number plant^-1^, biomass plant^-1^ and yield plant^-1^, but no significant effect on harvest index.

An unexpected disease (wheat stripe rust) during the 2019-2020 growing season led to a yield reduction in the F_2:3_ population. During the 2020-2021 growing season, no signs of disease were observed. Therefore, the effect of F_3:4_ on yield traits is closer to the actual effect of *Rht-yz*. The growth period of the Yanzhan mutation was 5-7 days later than that of Yanzhan, suggesting that *Rht-yz* may be involved in regulating the growth and development of wheat. Studies have also shown that *Rht4*+*Rht-B1b* has higher yield and harvest index under different moisture conditions (Liu et al., 2017). *Rht5* reduced wheat yield, but *Ppd-D1* effectively eliminated the negative effects of *Rht5* on yield and growth by advancing the growth period (Chen et al., 2018). These studies remind us that the negative effects of *Rht-yz* on yield can also be eliminated by introducing other new genes.

### *Rht-yz* improved wheat grain quality

Protein content, starch content and gluten content in wheat have an important impact on the promotion and application of wheat varieties. The quality of flour is mainly determined by protein and starch content (Ahmed, 2015). The dwarf gene *Rht-B1c* significantly increased the protein content, *Rht14* had a negative effect on quality traits (Duan et al., 2020b), and *Rht15* increased the protein and wet gluten content (Zhao et al., 2021). In this study, *Rht-yz* significantly increased crude protein, wet gluten and starch content. Therefore, *Rht-yz* is of great significance for improving the grain quality in wheat.

## Conclusions

In this study, a new dwarfing site, *Rht-yz*, was identified in wild mutants that could improve lodging resistance by reducing plant height, increasing basal internode diameter, wall thickness and mechanical strength, and had a positive effect on grain quality traits. In addition, *Rht-yz* had a negative effect on some yield traits. Therefore, the rational use of the dwarf gene *Rht-yz* has some utility for lodging resistance breeding in wheat, but attention should be paid to improving its negative effects.

## Data availability statement

The original contributions presented in the study are included in the article/**Supplementary Material**. Further inquiries can be directed to the corresponding author.

## Author contributions

JH: Investigation, Writing – original draft, Writing – review & editing. ZZ: Investigation, Writing – review & editing. XF: Investigation, Writing – review & editing. YZ: Investigation, Writing – review & editing. MA: Writing – review & editing. LM: Data curation, Writing – review & editing. YH: Data curation, Writing – review & editing. YL: Data curation, Writing – review & editing. YY: Data curation, Writing – review & editing. LZ: Data curation, Writing – review & editing. DS: Data curation, Writing – review & editing. CF: Writing – review & editing, Data curation. Y-GH: Funding acquisition, Supervision, Writing – review & editing. LC: Funding acquisition, Supervision, Writing – review & editing.
