# Supplementary material for: Effect of a novel dwarfing mutant site on chromosome 4B on agronomic traits in common wheat
Source: Front Plant Sci. 2024 Mar 20;15:1338425. doi: 10.3389/fpls.2024.1338425 (PMC10987870; doi:10.3389/fpls.2024.1338425)
Supplement: Supplementary file 1 [file Table_1.docx]

| **Name** | **Primer sequence** | **Tm（℃）** | **PCR production (bp)** |
| --- | --- | --- | --- |
| **PROF1** | TACCCCCTCCACACTTCTTCT | 59.5 | 1227 |
| **PROR1** | CGGGTGACACGTATACATAGCTAAT | 58.9 |  |
| **PROF2** | TCTTATGTTCGTCGTGCTCTTACT | 58.5 | 702 |
| **PROR2** | AGCTTTTGCTTGCCTCGATT | 58.0 |  |
| **GEF1** | CCCCCGACCCTGGATCCAAAT | 61.4 | 1553 |
| **GER1** | CAGGAATGTGCCGGAGTTGT | 59.9 |  |
| **GEF2** | AGCAGGAGGCGAACCACAACT | 63.7 | 639 |
| **GER2** | CTCTTCACGCCGGACGGTCGATC | 65.9 |  |

**SUPPLEMENT TABLE 1** Primer of genomic sequence clone.
